# Covid-19 Estimating the burden of symptomatic disease in the community and the impact of public health measures on physical, mental and social wellbeing: a study protocol

**DOI:** 10.12688/hrbopenres.13103.1

**Published:** 2020-08-03

**Authors:** M. Isabela Troya, Ali Khashan, Patricia Kearney, Ella Arensman, Philipp Hoevel, Claire Buckley, Margaret Fitzgerald, Rory Humphries, Elizabeth Kiely, Kieran Mulchrone, Mike Murphy, Lois O'Connor, Joan O'Donnell, Eilis O’Reilly, Micheal O'Riordain, Mary Spillane, Sebastian Wieczorek, Ivan J Perry

**Affiliations:** 1 School of Public Health, College of Medicine and Health, University College Cork, Cork, Ireland; 2 National Suicide Research Foundation, University College Cork, Cork, Ireland; 3 Australian Institute for Suicide Research and Prevention, School of Applied Psychology, Griffith University, Brisbane, Queensland, Australia; 4 School of Mathematical Sciences, University College Cork, Cork, Ireland; 5 Health Protection Surveillance Centre, Health Services Executive, Dublin, Ireland; 6 School of Applied Social Studies, University College Cork, Cork, Ireland; 7 School of Psychology, University College Cork, Cork, Ireland; 8 Department of Surgery, Mercy University Hospital, Cork, Ireland

**Keywords:** Virus diseases, public health, epidemiology, behaviour and behaviour mechanisms, mental health, mathematical modelling, network dynamics, non-pharmaceutical interventions

## Abstract

**Introduction**: Covid-19 was declared a pandemic in March 2020. Since then, governments have implemented unprecedented public health measures to contain the virus. This study will provide evidence to inform responses to the pandemic by: i) estimating population prevalence and trends of self-reported symptoms of Covid-19 and the proportions of symptomatic individuals and household contacts testing positive for Covid-19; ii) describing acceptance and compliance with physical-distancing measures, explore effects of public health measures on physical, mental and social wellbeing; iii) developing a mathematical network model to inform decisions on the optimal levels of physical distancing measures.

**Methods**: Two cross-sectional nationally-representative telephone surveys will be conducted in Ireland using random digit-dialling, with response rates estimates based on proportion of non-operational and non-answering numbers. The first survey with four waves in May and June will address adherence to social distancing measures and whether the respondent or other household members are or have been unwell during the preceding two weeks with one or more symptoms of Covid-19. The second survey with three waves in June, July and September will address knowledge, attitudes, and compliance towards physical-distancing measures and physical, mental and social wellbeing. The mathematical network model will be developed for all-Ireland (on various levels of spatial granularity including the scale of counties and electoral divisions) based on outputs from both cross-sectional surveys and relevant publicly available data to inform decisions on optimal levels and duration of physical distancing measures.

**Discussion**: This study will contribute to our understanding of the impact and sustainability of public health measures of the Covid-19 pandemic. Findings will have long-lasting benefits, informing decision-making on the best levels, and duration of physical-distancing measures, balancing a range of factors including capacity of the health service with the effects on individuals’ wellbeing and economic disruption. Findings will be shared with key policy-makers.

## Introduction

Covid-19 is a new illness caused by the SARS-CoV-2 virus. It was declared a pandemic by the World Health Organisation (WHO) in March 2020, only three months after the first registered case in Wuhan, China ( WHO, 2020). Since then, Covid-19 has rapidly spread, affecting 216 countries worldwide ( WHO, 2020). Covid-19 has similar symptoms to the common cold, spreading via respiratory droplets, and although in the majority of affected individuals’ symptoms range from mild to moderate, amongst vulnerable groups such as older adults and individuals with certain medical conditions, the virus can be deadly ( Rothan & Byrareddy, 2020). Covid-19 is a fast-moving illness, with different epicentres declared over the past few months, starting in Asia and then moving to Europe, North America and currently South America. Although most likely an underestimate due to limited public health capacities of countries worldwide, as of July 13, there are close to 13 million confirmed cases, and more than 565,000 confirmed deaths ( WHO, 2020). In Ireland, the first registered case was confirmed February 29, 2020.

In light of the rapid spread of the pandemic, governments worldwide have implemented unprecedented public health measures to contain the virus and mitigate its impact ( WHO, 2020). A variety of physical distancing measures including quarantine as well as restrictions on travel and social interactions have been implemented across the world. In Ireland, social restrictions started in March 2020, with a roadmap of easing of restrictions being implemented for a phased approach to the reopening of society ( Department of Health Ireland, 2020). These restrictions have been set in order to mitigate the effects of the virus, notwithstanding causing severe interruptions to various sectors including the education and economic sector, amongst others ( Douglas *et al.*, 2020). The effects of the public health measures taken, including physical restrictions, in combination with the impact and lethality of the virus are still unknown, but include serious short and long-term effects to physical, mental and social wellbeing of individuals ( Gunnell *et al.*, 2020; Holmes *et al.*, 2020). While there is evidence for the importance of quarantine on reducing incidence and mortality due to Covid-19 ( Nussbaumer-Streit *et al.*, 2020), and potentially avoiding second waves ( Prem *et al.*, 2020), data on the impact and sustainability of these restrictions is limited. Therefore, in order to inform national and global responses to the pandemic, it is critical to assess the impact and sustainability of the taken public health measures. This study aims to provide evidence to inform national and global responses to the Covid-19 pandemic. This study will:

i) Estimate population prevalence and trends of self-reported sensitive symptoms of Covid-19 and, via record linkage, the proportions of symptomatic individuals and their household contacts subsequently testing positive for Covid-19.
ii) Describe the acceptance and compliance with physical-distancing and related measures in the population, and explore the effects of the public health measures on the physical, mental and social wellbeing of individuals.
iii) Develop a mathematical network model to inform decisions on the optimal levels of physical distancing measures.

## Methods

This study protocol is reported in accordance with STROBE (The Strengthening the Reporting of Observational Studies in Epidemiology) guidelines for cross-sectional studies ( Von Elm *et al.*, 2014).

### Study design

The present study comprises multiple waves of data collection for two separate nationally-representative cross-sectional telephone surveys. The two surveys include some common questions on socio-demographics and adherence to public health measures with a separate focus on symptoms in the first survey and on mental health and social wellbeing in the second survey. Survey respondents who report symptoms will receive a follow-up call from a member of the research team to obtain a more detailed assessment of symptoms, timing of symptom onset and testing status.

A mathematical network model will be developed, informed by the data from the surveys and from other publicly available data.

### Setting

The study will be conducted in the Republic of Ireland. The four waves of the first telephone survey will be conducted on 1–14 May, 15–31 May, 2–14 June and 16–30 June. For Survey 2, the three telephone surveys will be carried out over a 2–3 week period in May, July and September 2020. The marketing company Ipsos MRBI will conduct all the telephone surveys in collaboration with researchers at University College Cork (UCC).

### Participants

Participants in the surveys will be selected from the general population. Eligibility criteria for the telephone surveys are: (a) adults aged 18 years and above, (b) residing in Ireland and having a landline or mobile telephone number. Participants will be excluded if they are 17 years or younger. In order to achieve a nationally representative sample, surveys will be conducted using random digit-dialling (approx. 80% mobile, 20% landline), with response estimates based on proportion of non-operational and non-answering numbers.

### Variables, data sources and measurement

***Survey 1.*** Survey 1 will examine sociodemographic variables, household composition, smoking and alcohol consumption, details of work and recent journeys outside the home by members of the household. Whether the respondent or other members of the household are currently unwell or have been unwell during the preceding two weeks with one or more sensitive symptoms for Covid-19 infection will also be queried (see Appendix 1, *Extended data* for Survey 1 Questions ( Troya, 2020)). Furthermore, individuals answering “yes” to the questions on Covid-19 symptoms among members of the household will be offered a follow-up call within 24–48 hours from a Public Health Medicine (PHM) physician. The PHM physician will take a short history on the relevant symptoms, as per the current standard of care for a symptomatic Covid-19 contact (including details of telephone number) and advise the individual to call their General Practitioner (GP) to arrange a test if appropriate and in line with national testing guidelines. While waiting for the test result, individuals with symptoms will be advised to self-isolate and family contacts will be advised to self-quarantine, as per current standard practice ( Health Protection Surveillance Centre, 2020). The PHM physician will obtain verbal consent (with assurances of confidentiality) for follow-up on Covid-19 test results using the National Computerised Infectious Disease Reporting (CIDR) information system in collaboration with the Health Protection Surveillance Centre, linking on name and date of birth. The PHM physician will also obtain contact details for the individual's GP and will call the GP to ask them to arrange Covid-19 testing for the individuals with symptoms and all close household contacts if appropriate. The relevant GPs will be advised that the patients and their household contacts reported symptoms as part of a national HPSC supported surveillance study, that we have obtained verbal consent for follow-up on their test results and that these findings will help estimate the underlying burden of disease in the community.

***Survey 2.*** Survey 2 includes a serious of internationally validated measures of physical, mental and social wellbeing as seen in Table 1 (see Appendix 2, *Extended data* for Survey 2 Questions ( Troya, 2020)). The adapted Zhong *et al.* (2020) scale will address knowledge about Covid-19 and attitudes to and compliance with control measures. Symptoms of depression and anxiety over the past two weeks will be verified with the 16-item Patient Health Questionnaire Anxiety-Depression Scale (PHQ-ADS) ( Kroenke *et al.*, 2016). The 12-item Social Wellbeing Index by Boreham *et al.* (2013) will assess participants’ social wellbeing. Questions relating to participants’ health (alcohol and tobacco consumption, health-seeking patterns) will also be included, in addition to wider socio-demographic questions (age, gender, region, social class, employment status, highest level of education). Lastly, the survey will include open-ended questions in relation to any adversity experienced within the last three and 12 months. From Wave 2 of the survey, the eight-item Woman Abuse Screening Tool, will be used to identify and assess intimate partner violence ( Brown *et al.*, 1996).

**Table 1.** Instruments included in Survey 2. Instruments included in Survey 2 on compliance with the public health measures and the impact of these measures on physical, mental and social wellbeing.

Data Items	Name of Instrument and authors	Number of Items	Applied in population based surveys
Knowledge about COVID-19 and adherence to control measures	Questionnaire of knowledge, attitudes, and practice towards COVID-19, adapted version ( Zhong *et al.*, 2020)	11	Yes
Symptoms of depression and anxiety over the past 2 weeks	Patient Health Questionnaire Anxiety-Depression Scale: PHQ-ADS ( Kroenke *et al.*, 2016)	16	Yes
Social wellbeing	Social Wellbeing Index ( Boreham *et al.*, 2013)	12	Yes
Stress and adversity	Open ended questions on stress and adversity in the last 3 & 12 months	2	Yes
Domestic violence (To be used from wave 2)	Women Abuse Screening Tool ( Brown *et al.*, 1996)	8	Yes

***Mathematical model.*** The modelling work will employ a meta-population model for all-Ireland with compartments for susceptible, infected, recovered, and quarantined ( Maier & Brockmann, 2020) and consider an additional class of deaths. Each meta-population refers to location, area or community and corresponds to one node in the proposed modelling framework. Theses nodes are connected by links indicating travel and commuting between different locations. This framework can be applied on various levels of spatial granularity and could easily be transferred to other countries. On the highest resolution, the network comprises 3440 electoral divisions (EDs) of the Republic of Ireland and 890 super output areas (SOAs) for Northern Ireland, which corresponds to local administrative units below the NUTS 3 regions, but coarser geographical areas like counties, will also be considered. We will numerically explore various scenarios including the five-phase roadmap for Ireland. In addition, we will investigate the effect of dynamic interventions that aim to keep the number of infected below a given threshold by dynamically adjusting containment measures on a national scale, which could also be implemented at a regional (county) or local (ED/SOA) level.

### Bias

For standardisation and ensuring best practice when conducting a telephone survey addressing sensitive topics and guiding respondents to relevant health and support services when required, virtual one-hour training workshops and debriefing sessions will be provided to the Ipsos interviewers by psychologists from the National Suicide Research Foundation (NSRF) and School of Applied Psychology, UCC (EA, MM, IT). These training workshops will be conducted up to one week before data collection commences for each survey.

### Study size

Survey 1 will include 950 participants in each of the four waves, resulting in 3,800 participants. Survey 2 will include 1,000 participants in each of the three waves, resulting in 3,000 participants. Taking into consideration response rates estimates based on proportion of non-operational and non-answering numbers, a sample size of 1,000 produces a two-sided 95% confidence interval with a width equal to 0.028 when the sample proportion is 0.05. 

### Statistical methods

Statistical software SPSS version 26 and Diver Solution (DivePort version 7.0) will be used to assist in the data analysis. Descriptive statistics will be used in order to summarise response data.

### Data management and access

All data will be safely stored and used in accordance with the Irish Data Protection Amendment Act of 2003 and General Data Protection Regulation (GDPR, 2018). Only anonymised data will be released in aggregate form in academic journal articles, conference papers, and reports. Existing protocols which are compliant with GDPR requirements will be followed by both UCC and Ipsos MRBI researchers to ensure participant confidentiality is maintained. No identifying information is requested in the telephone surveys, and participants are provided with a participant ID number. Once Ipsos interviewers complete the interviews, these will be securely sent to study members, who will be the only ones with access to the data. Information on participants’ responses will be securely stored electronically. Electronic data will be password-protected and stored on an encrypted computer or using a UCC supported online safe storage facility for ten years after collection, as required by regulations.

The code generated for the mathematical modelling will be made available on a shared platform and all publicly available data sets (e.g., Central Statistics Office and Northern Ireland Statistics and Research Agency), used in the models will be referenced.

### Ethical considerations and informed consent process

This study received ethical approval from the Clinical Research Ethics Committee of the Cork Teaching Hospital in April 2020 (Ref: EMC 4 (b) 05/05/20). Ethical aspects and participants’ wellbeing are considered throughout the study. There is a possibility that some of the interview questions may trigger emotional reactions in some respondents and guidance to appropriate services will be provided if required. To address this, workshops will be provided to the Ipsos interviewers by psychologists from the NSRF and the School of Applied Psychology (EA, MM, IT) prior to the start of the telephone surveys and debriefing sessions scheduled during the data collection phase. Furthermore, follow-up calls will be conducted by the psychology team when concerning cases are identified by interviewers, and participants have given consent for a follow-up call.

Prior to participants taking part in the survey, they will be asked orally for their consent. Given the implementation of this research during the Covid-19 pandemic, ethical approval was obtained for oral consent only, where participants would provide informed oral consent after being informed about the research by the interviewer and the online information sheet. Due to the nature of the pandemic, it was adequate not to obtain written informed consent as approved by the Clinical Research Ethics Committee of the Cork Teaching Hospital. At the start of every interview, respondents will be informed that responding to the survey questions is voluntary. At the start of every interview, respondents will also be advised that an information leaflet on the study prepared by the School of Public Health, UCC and a separate Privacy Notice prepared by Ipsos MRBI are available on both the website of the School of Public Health at University College Cork and the Ipsos MRBI website. These will include details that are necessary to ensure compliance with GDPR requirements. Instructions on how to locate these web resources will be provided and respondents will be informed that they may request copies of these documents by email or post. Copies of the information leaflet for participants are provided in Appendices 3 and 4 (see *Extended data* ( Troya, 2020)). If, upon reading the Information Leaflet or the Privacy Notice documents, a participant is not happy with any aspect of the research and makes this known, their survey details will be deleted. In the first survey, subjects in a household with one or more individuals reporting symptoms suggestive of Covid-19, who decline the offer of a telephone call from a PHM physician, will be advised by the interviewer to contact their GP.

### Patient and public involvement (PPI)

A patient who was one of the first cases of COVID-19 diagnosed and managed successfully in Ireland contributed to the initial design of this study and the decision to include the eight-item Woman Abuse Screening Tool in Wave 2 of Survey 2. The PPI partner will continue to be actively involved in the project.

### Study status

The four waves of the first survey have been completed. The first wave of the second survey has been completed and the second wave July 1, 2020. The mathematical modelling framework is currently developed and a first version is available as preprint on medRxiv ( Humphries, 2020). The current version of the code has been released as C++ library for modelling epidemics on networks, EpiGraph, via GitHub ( Humphries *et al.*, 2020).

## Discussion

Several policies and public health measures are currently being created and implemented in Ireland in order to mitigate the effects and impact of Covid-19. Ireland has continued to follow WHO guidance and recommendations ( WHO, 2020) where possible, on dealing with Covid-19, which include adapting a broad range of physical restrictions at a national level. These physical restrictions have been from restricting travel limits to 2 kilometres as of March 29 ^th^, increased to 5 kilometres on May 10 ^th^, and a further increase to 20 kilometres on June 14 ^th^. All of the surveys included in this research have been adapted to align with the travel restrictions in place at the time of the survey administration. Given the novelty of the virus, and fast-approaching impact on countries and health systems, there is currently no published evidence on the acceptance and compliance with physical-distancing and related measures in the Irish population. Furthermore, the effects of the public health measures on the physical, mental and social wellbeing of the Irish population remain unexplored. Findings from this study will contribute to building the evidence-base on the impact and sustainability of public health measures of the Covid-19 pandemic at a national level, which can be replicated and adapted globally. Moreover, findings from this survey will have long-lasting benefits through the creation of a mathematical model, which will inform decision making on the best levels, and duration of physical-distancing measures, balancing a range of factors including health service capacity, effects on individuals’ wellbeing and economic disruption.

### Dissemination of results

Results of this study will be summarised and published in peer-reviewed academic journals. Furthermore, following guidance from the HRB Covid-19 Rapid Response Funding Call (COV19 2020), all research outputs will be shared in a timely fashion with relevant stakeholders, which include but are not limited to the National Public Health Emergency Team and the National Health Protection Surveillance Centre (HPSC). Given that five of the study members and collaborators are actively working with the Health Service Executive (HSE) public health response team and HPSC, study members will be able to share the findings immediately. Due to the potential global impact of the study, findings of immediate relevance to the international response to the Covid-19 pandemic will also be forwarded to the WHO Headquarters Leadership Team in collaboration with colleagues on the National Public Health Emergency Team. Policy briefs for targeted policy-makers will be prepared in order to disseminate findings with key stakeholders. Lastly, findings from the surveys will inform the larger research programme and the creation of a mathematical model, optimising opportunity for effective public health decision making. These results will be broadly shared with key stakeholders as described previously.

## Data availability

### Underlying data

No underlying data are associated with this article.

### Extended data

Harvard Dataverse: Questionnaires for Surveys WP1 and WP2. https://doi.org/10.7910/DVN/EKUTFF ( Troya, 2020).

This project contains the following extended data:

- Survey 1 questionnaire in DOCX format (Appendix 1)
- Survey 2 questionnaire in DOCX format (Appendix 2)
- Survey 1 information sheet in DOCX format (Appendix 3)
- Survey 2 information sheet in DOCX format (Appendix 4)

Data are available under the terms of the Creative Commons Zero "No rights reserved" data waiver (CC0 1.0 Public domain dedication).
