# Supplementary material for: Covid-19 Estimating the burden of symptomatic disease in the community and the impact of public health measures on physical, mental and social wellbeing: a study protocol
Source: HRB Open Res. 2020 Aug 3;3:49. [Version 1] doi: 10.12688/hrbopenres.13103.1 (PMC7525335; doi:10.12688/hrbopenres.13103.1)
Supplement: Peer review history

# Peer review: Reviewer response for version 1

**DOI:** 10.21956/hrbopenres.14206.r27915

**Published:** 2020-09-29

**Authors:** Alex Almuedo Riera

**Affiliations:** 1 Department of International Health, ISGlobal, Barcelona Centre for International Health Research (CRESIB), Hospital Clínic-Universitat de Barcelona, Barcelona, Spain

This is a stimulating Study Protocol article where the researchers aim to provide evidence of public health response to COVID-19 pandemic specially concerning on his social impact and sustainability. The researchers target this objective by estimating population prevalence of self-reported symptoms of COVID-19 and its trends and providing data on acceptance and compliance of physical-distancing and related measures established for COVID-19 pandemic in Irish Population.

 Particularly of interest will be the development of a mathematical model that aims to apprise optimal public health decision on physical-distancing measures and its duration balancing factors related to health care system, individual mental and social wellbeing and economic effects.

 The study design is appropriate for the objectives described. The methodology of the study seems replicable nationally and globally by others researchers. Surveys of the study are available, rationalised and referenced. Data management and access is described however it does not appear that will be useable and accessible by others researches.

 One of the surveys includes items on woman abuse screening tools. It is important to highlight that questions about possible emotional reactions triggered by the study are address and explained in the protocol.

 Some minor comments:

- It would be desirable, if possible, to add COVID-19 or SARS-COV-2 and Ireland/Irish population/Europe/European population as Keywords.
- Although it is clear how one COVID-19 patient contributed to the first design of the study it is not clear how patient and public involvement will be managed.

Overall this study protocol is clearly exposed and it aims to share findings promptly. It is relevant that this study can provide evidence on physical, mental and social wellbeing effects of current public health measures to manage the present COVID-19 pandemic.

Is the study design appropriate for the research question?

Yes

Is the rationale for, and objectives of, the study clearly described?

Yes

Are sufficient details of the methods provided to allow replication by others?

Yes

Are the datasets clearly presented in a useable and accessible format?

Yes

Reviewer Expertise:

Tropical medicine and international Health, migrant health, emerging infectious diseases and High level isolation unit (UAAN).

I confirm that I have read this submission and believe that I have an appropriate level of expertise to confirm that it is of an acceptable scientific standard.

# Peer review: Reviewer response for version 1

**DOI:** 10.21956/hrbopenres.14206.r27900

**Published:** 2020-09-14

**Authors:** Ikenna Ebuenyi

**Affiliations:** 1 Assisting Living & Learning (ALL) Institute, Department of Psychology, Maynooth University, Maynooth, Ireland

Thank you for the opportunity to review this study protocol entitled: *‘Covid-19 Estimating the burden of symptomatic disease in the community and the impact of public health measures on physical, mental and social wellbeing: a study protocol’.*

 The rationale for and objectives of the study are justified and clear. The study would provide relevant data on COVID-19, perceptions about public health responses to COVID-19, and the perceived impact of these measures on wellbeing in Ireland. These findings and the anticipated mathematical model from the study may be useful in instituting evidence-based measures and responses for physical distancing in relation to COVID-19 and future pandemics.

 The study design is appropriate for the study objectives. The study methodology is comprehensive and may be replicated by interested researchers. The researchers have also made available four different files that contain information about the study and the questionnaire in a data repository. This is commendable and makes it easier for researchers interested in replicating the study to adapt the tools developed for this study.

 The datasets are presented in an accessible format. However, it is pertinent to highlight that although the authors declared that data is freely available in accordance with the ‘Creative Commons Zero “No rights reserved” data waiver’, the section on data access and management did not clearly confirm if data generated from the study will be available to other researchers and the condition(s) under which the data may be released. It is useful to clearly address the data availability statement.

 Overall, this study protocol is well written and would be an important addition to existing research on the management of COVID-19 in Ireland and globally.

Is the study design appropriate for the research question?

Yes

Is the rationale for, and objectives of, the study clearly described?

Yes

Are sufficient details of the methods provided to allow replication by others?

Yes

Are the datasets clearly presented in a useable and accessible format?

Yes

Reviewer Expertise:

Global Mental Health, Disability Inclusion, and Health System Research.

I confirm that I have read this submission and believe that I have an appropriate level of expertise to confirm that it is of an acceptable scientific standard.
